# Hyperspectral imaging meets 3D Gaussian Splatting: A novel approach beyond 3D plant morphology

**DOI:** 10.1016/j.plaphe.2026.100198

**Published:** 2026-03-12

**Authors:** Wenzhe Deng, Tingting Wu, Zhichao Ni, Yajie Liu, Hanyue Jia, Qingping Ling

**Affiliations:** a College of Mechanical and Electronic Engineering, Northwest A&F University, Yangling, 712100, China; b Shaanxi Key Laboratory of Agricultural Information Perception and Intelligent Service, Yangling, 712100, China; c Hainan Institute of Northwest A&F University, Sanya, 572025, China

**Keywords:** Plant phenotyping, Multimodal data fusion, Hyperspectral imaging, 3D Gaussian splatting, Hyperspectral point clouds

## Abstract

Accurate acquisition of plant phenotypes is crucial for elucidating plant growth and development, underlying genetic mechanisms, and responses to environmental stimuli. Traditional three-dimensional (3D) phenotyping mainly captures geometric traits such as height, leaf area, and canopy volume, while overlooking physiological and biochemical information. Here, we present a hyperspectral point clouds generation method based on PlantGaussian (a 3D Gaussian Splatting technique) that integrates structural and spectral information, extending 3D phenotyping beyond geometry to include physiology. High-quality plant point clouds were first reconstructed using PlantGaussian, and hyperspectral images(HSI) were mapped onto them to produce hyperspectral point clouds. In potted soybean experiments, we built predictive models linking hyperspectral reflectance to SPAD (chlorophyll content) and EWT (equivalent water thickness), and visualized their 3D distributions. The hyperspectral point clouds achieved strong predictive performance for SPAD (*R*^2^ = 0.78, *RMSE* = 2.05) and EWT (*R*^2^ = 0.80, *RMSE* = 1.07), thereby validating the approach. It further revealed clear vertical stratification within the canopy, highlighting significant spatial heterogeneity of SPAD and EWT in individual plants. Temporal monitoring from August 6 to 21, 2025, captured a sharp increase in EWT after heavy rainfall on August 11. Overall, our results demonstrate that hyperspectral point clouds enable accurate, non-destructive trait estimation and provide a powerful tool for exploring plant function, monitoring stress responses, and advancing precision agriculture.

## 1 Introduction

Plant phenotyping research is essential for understanding plant growth, development, the underlying genetic mechanisms, and responses to environmental changes [[1], [2], [3], [4]]. Although image-based methods for plant phenotypic trait extraction have been widely employed [[5], [6], [7], [8], [9], [10]], they inherently constrain the capacity to characterize the spatial and three-dimensional(3D) complexity of plants. This limitation restricts the comprehensive extraction of plant phenotypic information.

Advances in 3D phenotyping technologies offer promising solutions to overcome these challenges [[11], [12], [13]]. Specifically, unlike conventional 2D imaging, 3D plant phenotyping captures spatial structural information, thereby resolving issues such as leaf occlusion and enabling accurate quantification of traits inaccessible in two dimensions [[14], [15], [16], [17]]. This provides more comprehensive insights into plant structure and traits [[18], [19], [20]]. In particular, 3D Gaussian Splatting has emerged as a significant breakthrough in 3D reconstruction [21]. With brief training, it can render complex scenes in real-time and at large scale, requiring only sparse point clouds and camera poses as input. This means that operators simply need to capture a set of multi-view images around the plant, offering a highly promising method for high-fidelity 3D reconstruction of plants. Various macroscopic phenotypes can be extracted through mesh measurement and analysis, achieving high-fidelity 3D plant model construction and trait extraction. Many related studies have investigated plants from a 3D perspective to achieve more comprehensive phenotypic analyses [17,22]. However, approaches based solely on 3D plant models can only capture macroscopic parameters (e.g., leaf length, leaf inclination, etc.), Beyond macroscopic traits like height, leaf size, and shape, Physiological traits such as chlorophyll content, water status, photosynthetic rate, stomatal conductance, leaf temperature, and transpiration rate are essential for understanding genome-to-environment interactions [23,24].

To achieve a more comprehensive understanding of plant phenotypes, many studies have combined 3D imaging with spectral imaging technologies. Spectral measurements provide non-destructive insights into plant physiological status, including traits such as chlorophyll content, water status, and photosynthetic activity [[25], [26], [27]]. By integrating 3D structural information with spectral signals, these approaches enable accurate, spatially resolved quantification of plant traits, facilitating the assessment of growth dynamics, stress responses, and overall functional performance. Sun et al. [28] explored approaches for constructing multispectral point clouds of tomato plants in greenhouse environments. By registering multispectral images to corresponding depth data, they generated multispectral 3D point clouds, thereby facilitating the prediction of SPAD values Zhang et al. [29] also used a hyperspectral camera and a depth camera to generate hyperspectral point clouds, and further proposed a 3D calibration approach to improve the accuracy of spatial radiometric calibration in close-range hyperspectral imaging. Xie et al. [30] introduced a hemispherical-based calibration method for close-range multispectral images and generated multispectral point clouds by integrating multispectral and depth cameras. To address point cloud quality issues, they developed a next-best-view planning [31] to determine the optimal acquisition angles for generating high-quality point cloud data. Similarly, Zhu et al. [32] generated hyperspectral point clouds using both hyperspectral and depth cameras. However, point clouds generated by depth cameras are often not sufficiently dense and cannot reproduce plant morphology with high fidelity. These studies collectively demonstrate that integrating spectral data with point cloud data enables a more comprehensive representation of plant phenotypes. Compared with traditional 3D phenotyping, spectral point clouds are not limited to capturing macroscopic parameters but can also reveal physiological traits, such as chlorophyll and water content, that cannot be directly observed. This advantage arises from the ability of spectral techniques to provide a rapid, non-destructive means of acquiring both spatial and spectral data that reflect internal physiological characteristics [[33], [34], [35], [36]].

Previous studies have proposed various methods for generating spectral point clouds. In addition to the aforementioned approach of registering spectral images with depth images, Xiao et al. [37] reported the use of Structure-from-Motion (SfM) techniques to construct multispectral point clouds for investigating the 3D distribution of *Cercospora beticola* leaf spot in sugar beet. Spectral information was employed for disease identification, while the multispectral point clouds characterized the vertical and horizontal spatial distribution patterns of the disease, which is of great significance for understanding the 3D distribution and spread of plant diseases. However, related studies point out that plant point clouds created from images of different spectral bands possess complementary characteristics [38], which may cause spectral information loss at certain spatial locations for some bands. Therefore, directly generating spectral point clouds from multi-view spectral images may result in information loss. Elias et al. [39], in their study on building thermal imaging, proposed mapping RGB or thermal images onto existing point clouds to colorize them. Motivated by this, this study seeks to construct hyperspectral point clouds of plants by projecting hyperspectral information onto the reconstructed 3D point clouds models of plants.

Therefore, we propose a method for generating hyperspectral point clouds. In our previous work, we developed PlantGaussian [40], a framework that produces realistic 3D visualizations of plants across different time points and scenes by converting Gaussian rendering results into measurable plant meshes. Building upon this framework, HSI were mapped onto the sampled plant meshes (point clouds) based on voxel–pixel correspondences, thereby generating high-quality hyperspectral point clouds. To evaluate the effectiveness and application potential of the proposed hyperspectral point clouds generation method, we applied it to potted soybean plants for predicting SPAD and EWT values, which validated the capability of hyperspectral point clouds in 3D phenotypic analysis. In addition, temporal hyperspectral point clouds were collected, enabling long-term, continuous, and non-destructive monitoring of potted plants. Furthermore, we visualized the vertical spatial distributions of SPAD and EWT within soybean canopies to demonstrate the potential of hyperspectral point clouds for detailed physiological trait analysis. Overall, the proposed hyperspectral point clouds framework provides a powerful tool for comprehensive 3D plant phenotyping, as it enables the joint exploration of structural and physiological traits in 3D space, offering new opportunities for understanding plant function and monitoring stress responses.

## 2 Materials and methods

### 2.1 Data acquisition

**Fig. 1:**
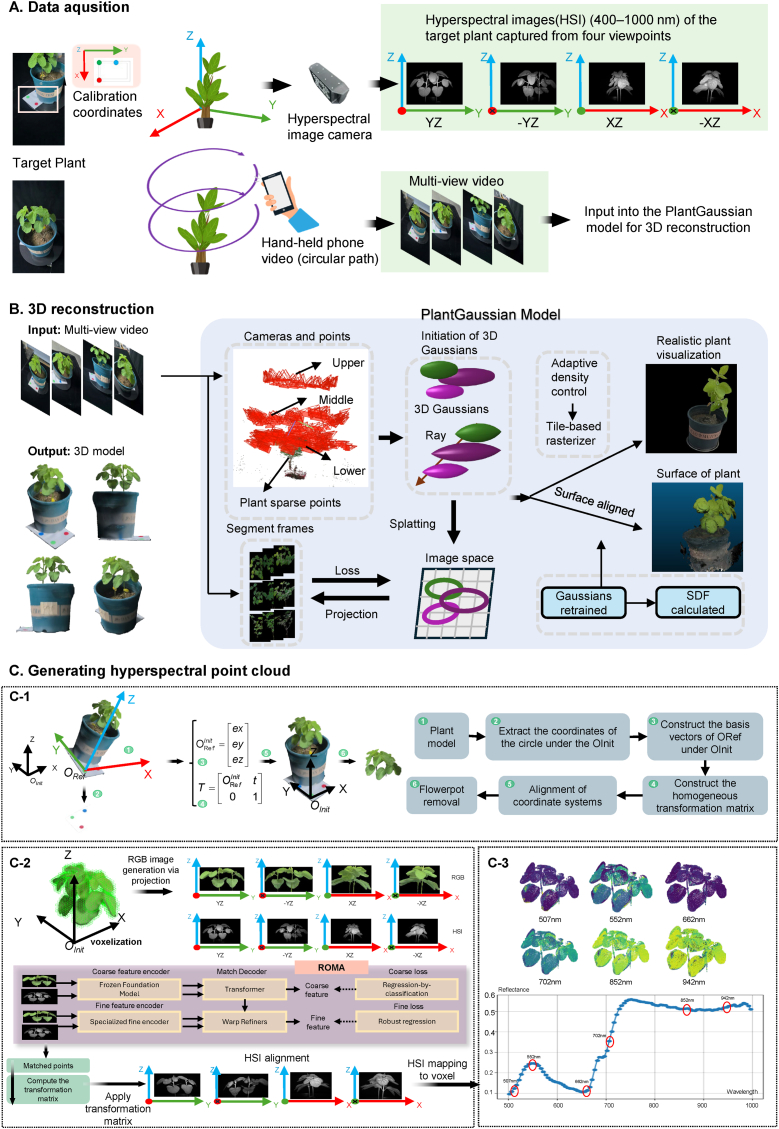
Pipeline of the approach A. Collect multi-view images of the target plant along with HSI from specific viewpoints. B. Reconstruct high-quality point clouds using PlantGaussian. C. Voxelize the high-quality point cloud and project it onto the viewpoints of hyperspectral image acquisition while recording the voxel–pixel correspondences. Then, register the HSI to the projected images, and map the hyperspectral data back onto the point cloud to generate a hyperspectral point clouds.

Experimental data were collected from August 6 to August 21, 2025. The data collection process comprised two components: multi-view and hyperspectral image acquisition, as illustrated in Fig. 1A. To ensure the continuity of multi-view images, Each plant was recorded along a circular path using a smartphone (frame rate 30 fps; resolution 1080 × 1920 pixels) for a duration of approximately 60 s. Following the method of PlantGaussian, we employed a uniform temporal sampling strategy to extract frames from the video, selecting one frame every 9 frames (approx. 0.3-s intervals). This process resulted in a dataset of approximately 200 images per plant. Given the 360° trajectory, the average angular spacing between adjacent frames was approximately 1.8°, ensuring sufficient overlap for high-quality 3D reconstruction. Furthermore, to guarantee data quality, we performed a manual visual inspection to identify and exclude any frames affected by motion blur prior to reconstruction. To facilitate spatial localization when mapping hyperspectral information onto the 3D plant model, a rigid right-angle board with red, green, and blue circular markers was placed beside each plant for spatial calibration.

HSI were collected using a handheld hyperspectral camera (HAIP BlackMobile, Germany). The HAIP BlackMobile is a portable visible–near-infrared hyperspectral camera with a spectral range of 502–997 nm across 100 spectral bands, enabling efficient and rapid hyperspectral image acquisition. The HAIP BlackMobile is capable of active illumination. Therefore, all data were acquired in a dark environment to ensure that the only light source was the active illumination provided by the HAIP BlackMobile. The plant remained stationary, while the camera was manually operated at an approximate distance of 0.5 m from the target. Prior to each acquisition, a polytetrafluoroethylene (PTFE) white reference panel with a reflectance of 0.95 was imaged and used as the reference for radiometric calibration. To ensure precise alignment, a rigid right angle calibration board was used to define an orthogonal coordinate system. The plant and the rigid right angle calibration board were placed on a high precision motorized rotation stage, while the hyperspectral camera remained fixed. By controlling the rotation stage, images were captured from four principal directions, 0°, 90°, 180°, and 270°. Although the acquisition was limited to only four viewpoints, this strategy, leveraging the dense geometric reconstruction provided by the PlantGaussian model, effectively covered most of the soybean canopy surface for spectral mapping. For each plant, HSI of four orthogonal planes defined by the rigid right-angle board were acquired.

Smartphone and hyperspectral imaging system settings are detailed in Table 1. After data collection, all datasets were processed in Python 3.8.

**Table 1:** Specifications and acquisition settings of RGB and hyperspectral imaging systems.

Device Category	Parameter	Specification/Setting
RGB Video Acquisition	Device	Smartphone (Huawei Mate 70)
	Acquisition mode	Auto
	Resolution	1920 × 1080 pixels
	Frame rate	30 fps
	Aperture	f/1.4
Hyperspectral Imaging	Spectral range	502–997 nm
	Spectral bands	100 bands
	Spatial resolution	640 × 480 pixels
	Spectral resolution	5 nm
	Integration time	5 ms
	Analog gain	3.0×
	Radiometric calibration	PTFE white reference panel (reflectance ∼0.95)
	Viewpoints	Four orthogonal viewing directions
	Object distance	0.5 m

### 2.2 Reconstructing 3D plants with PlantGaussian

To obtain high-fidelity and geometrically accurate 3D models, we employed PlantGaussian [40] for 3D reconstruction. As illustrated in Fig. 1B, PlantGaussian is a general-purpose plant 3D reconstruction model, previously reported by us, that is applicable across different scenes and time periods. By simply inputting a sequence of continuous multi-view images, PlantGaussian can reconstruct high-quality 3D plant models. In this study, we utilized the original configuration and default hyperparameter settings of PlantGaussian (trained for 30,000 iterations), as the data acquisition environment was consistent with the framework's design domain.

### 2.3 Generating hyperspectral point clouds

After reconstructing the plant model with PlantGaussian, we performed hyperspectral information mapping onto the point clouds (Fig. 1C). As illustrated in Fig. 1C-, we first aligned the initial coordinate system of the 3D model to a manually defined coordinate system using the reference board placed for spatial calibration. The alignment process was formulated as a rigid body transformation based on a change of basis in Euclidean space. Specifically, a standardized world coordinate system was defined according to the spatial configuration of the L-shaped calibration board. We designated the center of the green marker as the pivot point (origin). The normalized vector pointing from the green marker to the red marker defined the positive X-axis, while the vector from the green to the blue marker defined the positive Y-axis. The Z-axis was determined via the cross product of the X and Y basis vectors, ensuring a right-handed orthogonal system. Based on these unit vectors, we constructed a 4× 4 homogeneous transformation matrix, and its inverse was applied to the initial point cloud. This operation effectively mapped the plant model into the standardized reference frame, aligning the Z-axis with the plant's vertical growth direction. The detailed procedure is described in Algorithm 1.

Algorthm 1 Coordinate Alignment Using Calibration Board Markers

**Table:** 

**Input:** Initial point cloud $P={\left\{p_{i}\in R^{3}\right\}}_{i=1}^{N}$, marker positions **c**_*g*_, **c**_*r*_, **c**_*b*_
**Output:**Aligned point cloud $P^{\prime }$
Compute normalized X-axis direction: **u**_*x*_ = (**c**_*r*_ − **c**_*g*_)/‖**c**_*r*_ − **c**_*g*_‖
Compute normalized Y-axis direction: **u**_*y*_ = (**c**_*b*_ − **c**_*g*_)/‖**c**_*b*_ − **c**_*g*_‖
Compute Z-axis to form a right-handed coordinate system: **u**_*z*_ = **u**_*x*_ × **u**_*y*_
Construct rotation matrix: **R** = [**u**_*x*_**u**_*y*_**u**_*z*_]
Form homogeneous transformation matrix: $T=\left[\begin{array}{ll} R & 0\\ 0^{⊤} & 1 \end{array}\right]$
Apply inverse transformation to each point: ${\tilde{p}}_{i}^{\prime }=T^{-1}{\tilde{p}}_{i}$
Return aligned point cloud $P^{\prime }$

**Fig. 2:**
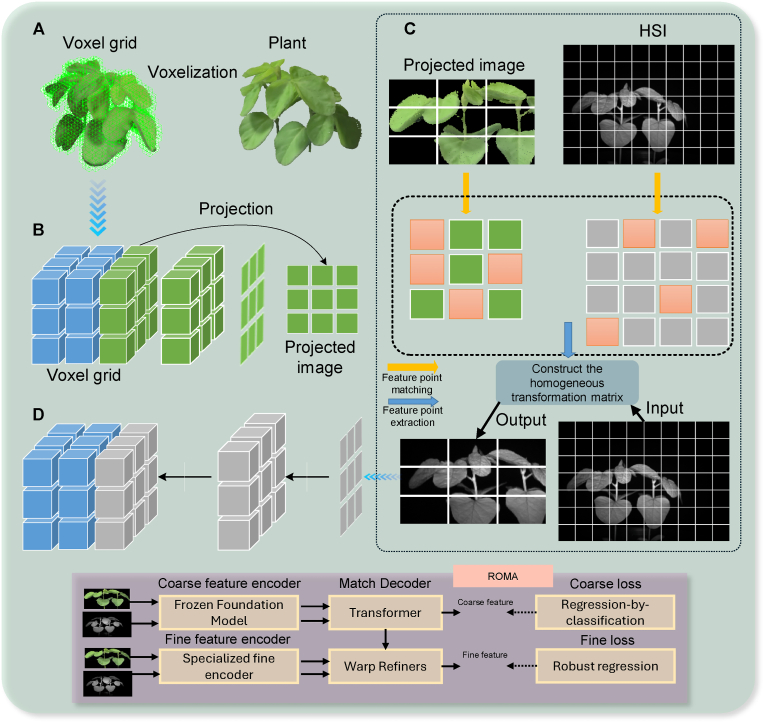
Generation of hyperspectral point clouds A. Voxelization of the reconstructed 3D plant model B. Project the voxel grid onto the viewpoint of the acquired hyperspectral images, and record the correspondence between voxel indices and pixel indices C. Spatially align the HSI to the projected images. D. Remap the spatially aligned HSI back onto the plant voxel grid according to the voxel-pixel correspondence.

**Fig. 3:**
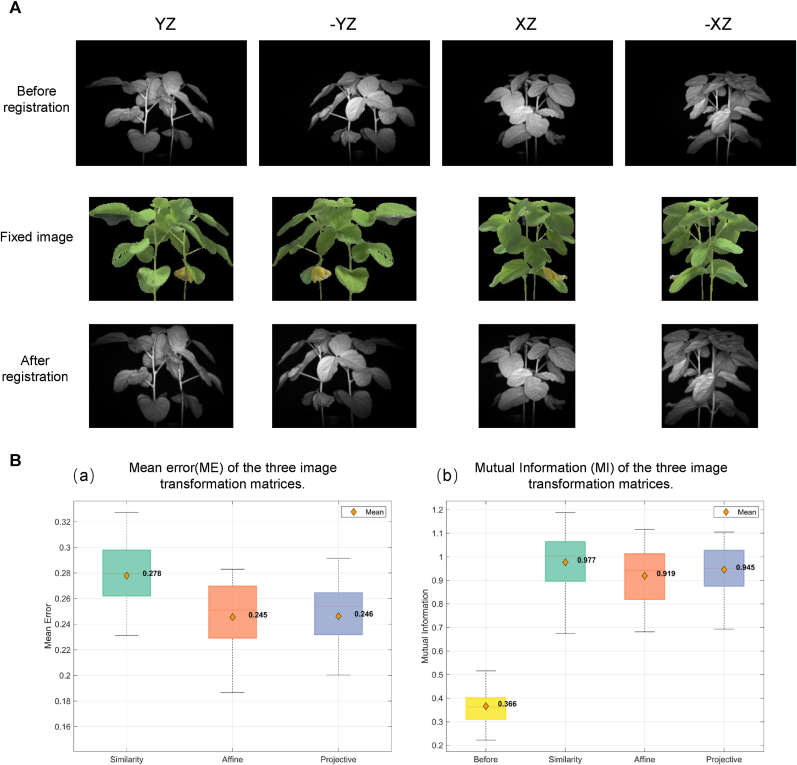
Registration results of the hyperspectral image to the projection image.

The point clouds was then voxelized and projected onto four orthogonal planes of the aligned coordinate system to generate color images. During this projection process, the correspondence between voxels and the pixels of the generated color images were recorded (Figs. 1C–2). Subsequently, HSI acquired from the four orthogonal planes were registered to the projection images. Finally, based on the voxel–pixel correspondence recorded during the projection, the registered hyperspectral information was mapped back onto the point clouds, resulting in a hyperspectral point clouds. Figs. 1C–3 presents the spectral point clouds of six bands along with the corresponding spectral curves.

#### 2.3.1 Voxel grid generation

Voxelization of the plant 3D model is the process of converting a continuous geometric model into a discretized 3D voxel grid [41], as shown in Fig. 2A. This method divides the plant model into regular cubic units (voxels), each assigned a unique spatial index by discrete coordinates (*i*, *j*, *k*). The corresponding voxel can be easily located based on the index. Compared to unstructured point cloud data, voxelization reduces computational overhead. During voxelization, the color information of each voxel is obtained by averaging the colors of all points within the voxel center. The above processing is implemented in Python 3.8 using the Open3D library. An adaptive resolution strategy was adopted to maintain high reconstruction fidelity across plants of varying sizes. Specifically, a target resolution factor of *N* = 512 was defined. For each individual plant, the voxel size (spacing) was dynamically computed by dividing the maximum dimension of the plant's three-dimensional bounding box by this resolution factor. Voxelization was performed using the Open3D library. This adaptive formulation ensures that a consistent level of geometric detail is preserved regardless of plant scale.

#### 2.3.2 Projecting the point clouds

To establish a geometric mapping between the 3D voxel representation and the 2D hyperspectral images, a voxel projection method is constructed, projecting the 3D voxel grid onto 2D planes along multiple orthogonal directions. As illustrated in Fig. 2B, the voxel grid can be projected onto planes such as YZ, XZ according to predefined acquisition directions. Each voxel visible along the viewing direction is represented as a pixel in the projection image, thereby establishing a mapping between the 3D voxels and the 2D images.The spatial resolution of these projected two dimensional images is determined by the voxel grid dimension parameter *N*. In our experimental setup, *N* was set to 512, resulting in a maximum projection image resolution of 512 pixels. This resolution was selected to match the spatial resolution of the hyperspectral images, thereby ensuring scale consistency in the subsequent image registration steps while maintaining computational efficiency. The voxel grid was constructed according to the true proportions of the original plant, and all projections were performed based on the relative geometric proportions of the real plant. In this process, all voxels visible from the projection direction are recorded in the image, effectively capturing the 3D structural information perceived from that viewpoint.

To formalize this process, the 3D voxel grid is first represented as a finite discrete index set:

(1) $$V\in Z^{3}$$

Each voxel can be represented as:

(2) $$\left(i,j,k\right)\in V,i\in \left[0,N_{x}\right),j\in \left[0,N_{y}\right),k\in \left[0,N_{z}\right)$$

where *i*, *j*, and *k* represent the voxel indices along the *x*, *y*, and *z* directions, respectively, and *N*_*x*_, *N*_*y*_, and *N*_*z*_ denote the 3D dimensions of the voxel grid.

The process of projecting the voxel grid onto a specific direction to generate an image can be defined as a projection mapping function *F*, which maps the 3D voxel indices to 2D image indices. The *YZ* plane is taken as a representative example, the function is defined as follows:

(3) $$F_{yz}:V\to Z^{3},F_{yz}\left(i,j,k\right)=\left(i,j\right)$$

Similarly, the projection functions onto *XZ* planes are defined as:

(4) $$F_{xz}\left(i,j,k\right)=\left(j,k\right)$$

The aforementioned projection functions ignore the depth information of voxels along the projection dimension. Therefore, to ensure that occluded voxels are not incorrectly projected onto the image when viewed from a specific direction, a deterministic depth selection mechanism is introduced.

Specifically, during the voxel projection process, a depth selection function is introduced for each 2D projection coordinate to uniquely select the voxel closest to the viewing point at that projection position. Taking the projection onto the *YZ* plane as an example, if the viewpoint is located in the +*X* direction, the voxel with the largest *k* value at the same (*i*, *j*) should be selected; if the viewpoint is in the −*X* direction, the voxel with the smallest *k* value is selected. The depth selection function can be defined as follows:

(5) $$\left\{\begin{array}{l} k^{*}=\arg\max_{k}\left\{k|\left(i,j,k\right)\in V\right\}\\ k^{*}={\mathrm{arg min}}_{k}\left\{k|\left(i,j,k\right)\in V\right\} \end{array}\right)$$

Accordingly, the set of image pixels generated by projecting onto the *YZ* plane can be formalized as:

(6) $$I_{yz}\left(i,j\right)=F_{yz}\left(i,j,k^{*}\right)\in R^{N_{y}\times N_{z}}$$

where *i* and *j* represent the pixel indices in the 2D image.

To establish a unique correspondence between voxels and image pixels, a lookup table (LUT) is constructed for each 2D pixel during the projection process to record its corresponding 3D voxel index:

(7) $$M_{yz}\left(i,j\right)=k^{*}\Rightarrow \left(i,j,k^{*}\right)$$

The LUT mechanism is also applied to projections in the *XZ* directions. During the orthogonal projection image generation process, corresponding mappings between pixels and voxel indices are established, providing the necessary structural support for mapping image pixel information back to 3D space. Due to the determinism of the projection and depth selection functions, each image pixel has a complete one-to-one correspondence with a 3D voxel, ensuring invertibility and geometric consistency in subsequent processing. This mapping guarantees that every pixel in the projection image uniquely corresponds to a voxel in the voxel grid, laying the foundation for subsequent hyperspectral information registration and fusion.

#### 2.3.3 Registration of hyperspectral and projected images

In this study, an image registration framework was developed based on RoMa (Robust Dense Feature Matching) [42] feature extraction and multi-model transformation. As shown in Fig. 2C, the green channel of the projection image was selected as the fixed image [30], since within the visible spectrum the green channel typically provides good contrast and clear edge information, which facilitates feature extraction and matching. Correspondingly, in the hyperspectral image, the band at 742 nm in the near-infrared range was chosen as the registration input. At this wavelength, the soybean canopy exhibits strong structural and textural variations, while avoiding the saturation effects that may occur in some visible bands. In our preliminary experiments, multiple spectral bands were evaluated, and the 742 nm band consistently produced more stable and reliable feature matching when used with the RoMa framework.

The registration between the hyperspectral image and the projected image is performed using the RoMa framework, which operates in a two-stage manner. In the first stage, coarse feature matching is employed to establish global correspondences between the hyperspectral image *I*_HSI_ and the projected image *I*_proj_. In the second stage, fine feature matching is conducted to refine the alignment and achieve sub-pixel registration accuracy. The feature extraction process can be expressed as:

(8) $$\left\{\varphi_{\text{coarse}}^{\text{HSI}},\varphi_{\text{fine}}^{\text{HSI}}\right\}=F_{\theta }\left(I_{\text{HSI}}\right),\;\left\{\varphi_{\text{coarse}}^{\text{proj}},\varphi_{\text{fine}}^{\text{proj}}\right\}=F_{\theta }\left(I_{\text{proj}}\right),$$

where *F*_*θ*_ denotes the two-stream feature encoder.

Following the RoMa framework, coarse features *φ*_coarse_ are obtained from frozen DINOv2 [43] representations, which provide robust and semantically consistent global context, while fine features *φ*_fine_ are extracted from a specialized ConvNet backbone to enhance spatial precision and local discriminability.

Based on these feature representations, the coarse global correspondences are estimated by a transformer-based matcher *G*_*θ*_ that predicts anchor probabilities through a regression-by-classification formulation:

(9) $$M_{\text{coarse}}=G_{\theta }\left(\varphi_{\text{coarse}}^{\text{HSI}},\varphi_{\text{coarse}}^{\text{proj}}\right).$$

Subsequently, a refinement module *R*_*θ*_ performs robust regression to locally adjust the coarse warp $M_{\text{coarse}}$, producing a fine-level correspondence map $M_{\text{fine}}$:

(10) $$M_{\text{fine}}=R_{\theta }\left(\varphi_{\text{fine}}^{\text{HSI}},\varphi_{\text{fine}}^{\text{proj}},M_{\text{coarse}}\right).$$

Dense pixel-level correspondences were finally obtained from the RoMa model, and a confidence threshold (>0.99) was applied to remove unreliable matches. To ensure robustness against outliers, the RANSAC algorithm [44] was employed to iteratively sample and validate the correspondences, retaining only a consistent set of inliers for subsequent geometric estimation. During the transformation model estimation stage, three geometric models (similarity, affine, and homography) were estimated in parallel based on the inlier set. For each model, the mean reprojection error was computed, and the model with the lowest error was automatically selected as the optimal registration solution.

In addition to geometric consistency, this study also incorporates Mutual Information (MI) as a complementary statistical metric to evaluate image registration performance. As a measure of statistical dependence between images, mutual information has been widely used in multimodal image registration tasks [45]. By integrating geometric accuracy with statistical consistency, a more comprehensive and objective assessment of registration quality can be achieved, ensuring reliable alignment between hyperspectral and projected images in both spatial structure and radiometric characteristics.

#### 2.3.4 Mapping hyperspectral information onto the voxel grid

After completing the spatial registration between the hyperspectral image and the projection image, the next step is to accurately map the spectral information from the hyperspectral image back to the 3D voxel grid. As illustrated in Fig. 2D, the core of this process lies in establishing a precise one-to-one correspondence between each pixel in the hyperspectral image and the corresponding voxel cell, thereby enabling the effective association of hyperspectral data with the 3D voxel structure. Specifically, the registration ensures spatial alignment between the projection image and the hyperspectral image, providing a consistent spatial reference for subsequent mapping. Based on this alignment, the voxel–pixel LUT established by PlantGaussian during the projection stage is employed to perform inverse mapping, determining the voxel position corresponding to each hyperspectral pixel. The spectral information of each registered pixel is then assigned to its corresponding voxel cell. When multiple hyperspectral pixels from different viewpoints are mapped to the same voxel, their spectral values are aggregated using a simple averaging strategy. This choice is motivated by the controlled acquisition setup and the high geometric consistency ensured by the RoMa-based registration framework. The one-to-one relationship established during the projection stage guarantees that hyperspectral information is correctly mapped back into the voxel grid, ultimately generating the hyperspectral point clouds. A direct quantitative evaluation of hyperspectral-to-voxel mapping accuracy is not performed, as voxel-level ground-truth hyperspectral references are not available. Instead, the effectiveness of the mapping is indirectly reflected by the subsequent registration accuracy and visual consistency of the generated hyperspectral point clouds.

Since the mapping principles for the XZ and YZ planes are identical, the YZ plane is used here as an illustrative example. Let the registered hyperspectral image be denoted as *I*_HSI_(*u*, *v*, *λ*), where (*u*, *v*) represents the spatial coordinates and *λ* the wavelength index. The 3D voxel grid is expressed as *V*(*i*, *j*, *k*), with (*i*, *j*, *k*) representing the discrete voxel indices along the *x*, *y*, and *z* axes, respectively. During the projection stage, corresponding LUT *M*_yz_ was recorded. Based on the established LUT, the mapping from the hyperspectral image to the voxel grid can be formulated as:

(11) $$\left(i,j,k\right)=M_{\text{yz}}\left(u,v\right),$$

where *M*_yz_ denotes the inverse voxel–pixel mapping.

For each hyperspectral pixel (*u*, *v*), its spectral vector is defined as:

(12) $$s\left(u,v\right)=\left[I_{\text{HSI}}\left(u,v,\lambda_{1}\right),\ldots ,I_{\text{HSI}}\left(u,v,\lambda_{N}\right)\right].$$

The spectral vector **s**(*u*, *v*) is then assigned to its corresponding voxel cell (*i*, *j*, *k*) according to the inverse mapping relationship:

(13) $$V\left(i,j,k\right)\leftarrow s\left(u,v\right),\;\text{for}\;\left(i,j,k\right)=M_{\text{yz}}\left(u,v\right).$$

This process ensures that each voxel inherits its corresponding spectral signature from the hyperspectral image. Since the LUT *M*_yz_ is deterministically constructed during projection, the inverse mapping is guaranteed to be one-to-one and invertible, ensuring geometric consistency and preserving the spatial correspondence between voxels and pixels.

### 2.4 Experimental setup

To validate the effectiveness and application potential of the proposed method in 3D phenotypic analysis, we collected a dataset of potted soybeans for generating hyperspectral point clouds. The soybean variety used in this study was Zhonghuang 13 (*Glycine max* L. Merr.). The plants were cultivated outdoors in pots at the Northwest A&F University, Yangling, Shaanxi Province, China(34°28′N, 108°07′E), to expose them to natural environmental conditions. The soybeans were planted on July 28, 2025, and both multi-view RGB videos (for 3D reconstruction) and HSI were collected synchronously every two days from August 6 until August 21, 2025, to generate time-series hyperspectral point clouds.

After the final on August 21, SPAD and EWT measurements were conducted for each leaf, Specifically, a total of 96 leaves were collected from 7 soybean plants (approximately 12 leaves per plant), ensuring a representative distribution from the top to the bottom of the canopy. These measurements provided a dataset for model training, while the hyperspectral data collected on previous dates (August 6–18) were reserved exclusively for applying the trained models to monitor temporal traits. A handheld SPAD meter (Konica Minolta, Japan) was used to take measurements at three evenly distributed points on each leaf, and the mean of these three readings was recorded as the SPAD value of that leaf. Measurements were performed for all leaves of each soybean plant. Subsequently, all leaves were excised and their fresh weight was measured. The leaves were then spread on a black velvet background for imaging to determine leaf area. Next, the leaves were oven-dried at 105°C for 30 min to deactivate enzymes, followed by drying at 70°C to constant weight to record dry weight. Leaf water content was calculated as the difference between fresh and dry weight, and EWT was obtained by dividing water content by leaf area. The calculation formula [46] for EWT is as follows:

(14) $$EWT=\frac{FW-DW}{LA}$$

where *FW* is the fresh weight, *DW* is the dry weight, and *LA* is the leaf area.

For modeling, the mean hyperspectral signature of each leaf(covering all 100 spectral bands from 502 to 997 nm) was extracted from the hyperspectral point clouds and used as an input variable to predict SPAD and EWT. Notably, taking advantage of the 3D structure provided by hyperspectral point clouds, singular value decomposition (SVD) was applied to compute the normal vector of each leaf, which was incorporated as an additional feature. Multi-block partial least squares regression (MB-PLSR) was then employed to construct predictive models. This method partitions multi-source data into functional or structural blocks and quantifies the contribution of each block to the model [47,48]. Specifically, the block importance was calculated based on the squared block weights corresponding to the selected latent variables, reflecting the relative amount of variance in the dependent variable explained by each data block. In this study, leaf hyperspectral data were defined as the *X*1 block, and leaf normal vectors as the *X*2 block. Both blocks were jointly used as inputs to the MB-PLSR model to predict SPAD and EWT. The analysis was conducted in Python 3.8. Regarding data preprocessing, the hyperspectral data block (*X*_1_) was first transformed using standard normal variate (SNV) to mitigate scattering effects caused by leaf geometry. Subsequently, both the predictor variables (*X*_1_: spectral features; *X*_2_: normal vectors) and the response variables (*Y*: SPAD/EWT) were mean-centered and scaled to unit variance. This step ensures that structural and spectral data with different physical units contribute comparably to the regression model. The optimal number of latent variables (LVs) was determined using leave-one-out cross-validation (LOOCV) by minimizing the cross-validated root mean square error (RMSECV) over a search range of 1 to 40 LVs. Model performance was evaluated using *R*^2^, RMSE, and MAE, and the contributions of hyperspectral features and leaf normal vectors to the models were quantified. The calculation formulas for *R*^2^, RMSE, and MAE are as follows:

(15) $$R^{2}=1-\frac{\sum_{i=1}^{n}{\left(y_{i}-{\hat{y}}_{i}\right)}^{2}}{\sum_{i=1}^{n}{\left(y_{i}-\bar{y}\right)}^{2}}$$

(16) $$RMSE=\sqrt{\frac{1}{n}\overset{n}{\underset{i=1}{\sum }}{\left(y_{i}-{\hat{y}}_{i}\right)}^{2}}$$

(17) $$MAE=\frac{1}{n}\overset{n}{\underset{i=1}{\sum }}|y_{i}-{\hat{y}}_{i}|$$

where *y*_*i*_ is the observed value, ${\hat{y}}_{i}$ is the predicted value, $\bar{y}$ is the mean of the observed values, and *n* is the number of samples.

Finally, the established models were applied to the time-series hyperspectral point clouds data collected between August 6 and August 21, 2025, enabling dynamic visualization of SPAD and EWT, thereby demonstrating the application potential of the proposed method.

## 3 Results

### 3.1 Evaluation of image registration

A visual comparison of the HSI acquired from four orthogonal directions and the projection image (fixed image) before and after registration is provided in Fig. 3A. Prior to registration, the HSI were inconsistent with the projection image in terms of size and spatial position. After registration, the HSI became spatially aligned with the projected RGB image, establishing a one-to-one correspondence. This spatial consistency enables the HSI to be mapped back into the point cloud using the voxel–pixel LUT, thereby generating the hyperspectral point clouds.

Quantitative evaluation results are summarized in Fig. 3B(a), which reports the mean registration error and mutual information (MI) metrics. In the mean error statistics, similarity, affine, and projective transformations all achieved small errors, each below 0.5 pixels, indicating the effectiveness of the proposed registration workflow. The average errors obtained by the three transformation types were 0.278, 0.245, and 0.246, respectively. These errors likely arise from rotations, translations, or tilts introduced during hyperspectral image acquisition relative to the orthogonal planes. Our registration approach addresses this by computing similarity, affine, and projective transformations based on matched keypoints between the moving and fixed images, and then selecting the transformation with the lowest mean error, which ensures the robustness of the registration process.

The changes in mutual information between hyperspectral and projection images are further illustrated in Fig. 3B(b). All three transformation types produced significant improvements in MI, with the mean value increasing from 0.366 to 0.977, 0.919, and 0.945, respectively. Overall, the proposed registration method demonstrates excellent performance and meets the requirements of the image registration step in our hyperspectral point clouds generation approach.

### 3.2 Visualization of the hyperspectral point clouds

**Fig. 4:**
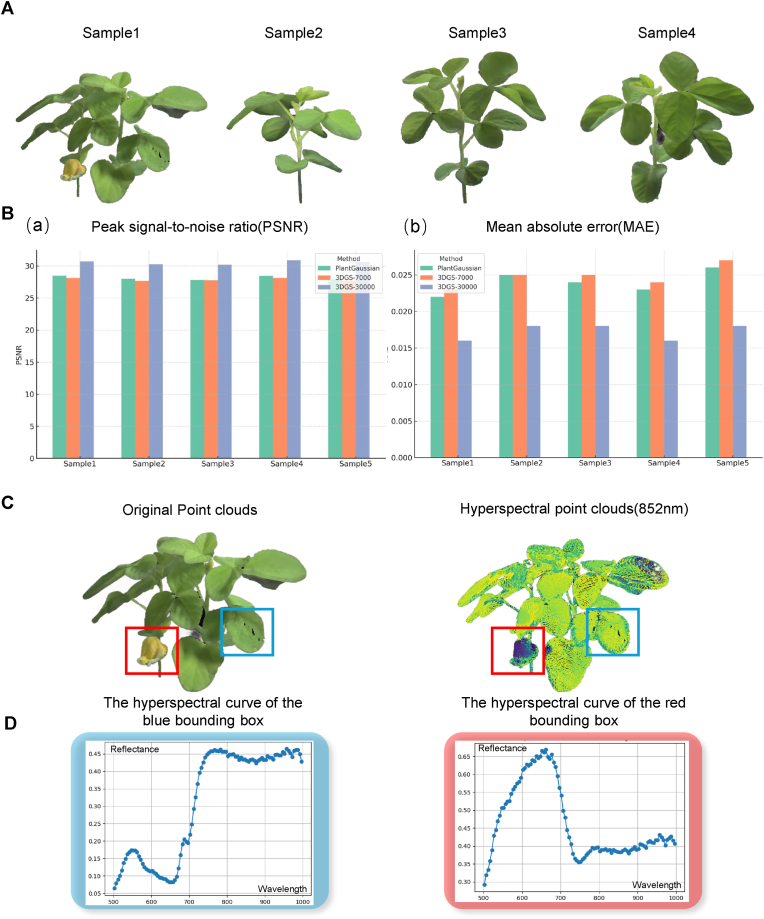
3D Reconstruction and Hyperspectral Point Clouds Generation A. 3D Reconstruction B. 3D Reconstruction Performance Evaluation C. Hyperspectral Point Clouds D. Hyperspectral curve.

Fig. 4A presents the results of 3D reconstruction of soybean plants using PlantGaussian, which achieved excellent performance in plant reconstruction tasks.

A comparative evaluation is provided in Fig. 4B, where PlantGaussian is benchmarked against 3D Gaussian at 7000 iterations (3DGS-7000) and 30,000 iterations (3DGS-30000), using peak signal-to-noise ratio (PSNR) and mean absolute error (MAE) as the evaluation metrics. For PSNR across the four displayed samples, PlantGaussian achieves nearly the same performance as 3DGS-7000 but remains significantly lower than 3DGS-30000. In terms of MAE, PlantGaussian also outperforms 3DGS-7000 but does not reach the performance of 3DGS-30000. This discrepancy arises because 3DGS approaches its optimal performance only after 30,000 iterations. However, the outputs of 3DGS are produced by a dedicated renderer, making them non-measurable and unsuitable for 3D plant phenotyping, whereas PlantGaussian balances both high fidelity and geometric measurability of plant structures.

Fig. 4C shows the generated hyperspectral point clouds, with the 852 nm band selected for visualization. It can be clearly observed that the yellowed leaf region highlighted by the red bounding box exhibits lower reflectance at 852 nm, indicating that the hyperspectral point clouds effectively captures this physiological condition. The 852 nm band lies within the near-infrared region of the spectrum, where yellowed leaves, due to water loss and degradation of cellular structure, typically show reduced reflectance.

We further extracted spectral curves from the red and blue boxed regions in Fig. 4C–as shown in Fig. 4D. Healthy leaves follow the typical reflectance pattern of green foliage: a distinct peak in the green region, a sharp increase at the red edge, and strong reflectance in the near-infrared. In contrast, yellowed leaves exhibit lower reflectance in the near-infrared region, while reduced chlorophyll content causes an increase in reflectance within the visible spectrum. These spectral characteristics of leaves are faithfully captured by the hyperspectral point clouds, thereby validating the effectiveness of our hyperspectral point clouds generation approach.

### 3.3 Modeling of phenotypic traits

**Fig. 5:**
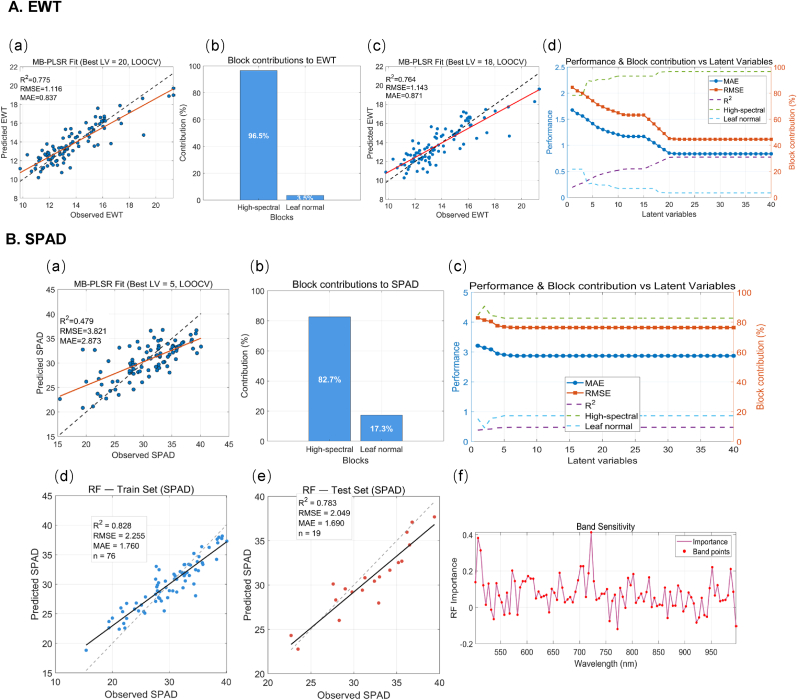
Prediction model results.

In Fig. 5, we present the modeling results of SPAD and EWT using MB-PLSR. Notably, when using both hyperspectral data and leaf normal vectors as input variables, MB-PLSR achieved relatively good results for EWT, whereas its performance for SPAD was only moderate. Therefore, we further supplemented SPAD modeling with a Random Forest approach.

Fig. 5A(a) shows the results of modeling EWT with MB-PLSR. During the modeling process, leave-one-out cross-validation was applied to evaluate model performance, and the optimal number of latent variables was determined as 20. The results showed that *R*^2^ = 0.775, *RMSE* = 1.116, and *MAE* = 0.837, indicating that the MB-PLSR model performed well. Fig. 5A(b) illustrates the contribution of each data block (hyperspectral data and leaf normal vectors) under the optimal latent variable setting. We found that leaf normal vectors contributed only 3.5% to the model, while hyperspectral data accounted for 96.5%. This may be because leaf normal vectors mainly describe the spatial orientation of leaves, whereas EWT primarily reflects internal water content, making the contribution of normal vectors relatively limited. Fig. 5A(c) presents the results of modeling with hyperspectral data alone, which achieved comparable performance to the model that included leaf normal vectors(*R*^2^ = 0.764), *RMSE* = 1.143, and *MAE* = 0.871). Fig. 5A(d) further depicts the curves of *R*^2^, *RMSE*, *MAE*, and block contributions as the number of latent variables increased. It is worth noting that, regardless of latent variable count, hyperspectral data consistently contributed far more to the model than leaf normal vectors.

Fig. 5B(a) presents the results of SPAD modeling with MB-PLSR. Using leave-one-out cross-validation, we determined the optimal number of latent variables to be 5. The results showed that *R*^2^ = 0.479, *RMSE* = 3.821, and *MAE* = 2.873, indicating that MB-PLSR did not achieve good performance for SPAD. This may be because SPAD is sensitive to only a few spectral bands, while redundancy among hyperspectral bands introduces noise, thereby reducing model accuracy. In Fig. 5B(b), the contribution of leaf normal vectors to the model reached 17.3%, whereas hyperspectral data contributed 82.7%. Although the relative contribution of normal vectors to SPAD prediction increased compared with EWT, it still remained much lower than that of hyperspectral data. Fig. 5B(c) shows the variation of *R*^2^, *RMSE*, *MAE*, and block contributions as the number of latent variables increased. Compared with EWT, the contribution of leaf normal vectors to SPAD prediction was higher, but overall still lower than that of hyperspectral data.

To further validate the effectiveness of hyperspectral data for SPAD modeling, we employed a Random Forest model using hyperspectral data. The dataset was split into training and testing sets at an 8:2 ratio. The results demonstrated that for the training set(Fig. 5B(d)), *R*^2^ = 0.828, *RMSE* = 2.255, *MAE* = 1.760; and for the testing set(Fig. 5B(e)), *R*^2^ = 0.783, *RMSE* = 2.049, *MAE* = 1.690. Fig. 5B(f) further evaluated the band importance curve during the modeling process, showing that only a few specific bands were highly sensitive to SPAD.

### 3.4 Temporal dynamics of phenotypic traits

**Fig. 6:**
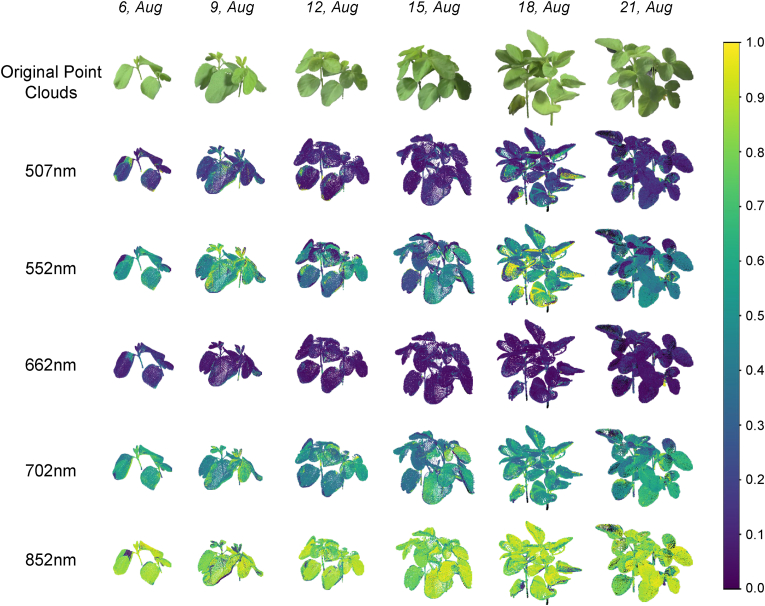
Temporal hyperspectral point clouds.

We applied the trained trait-prediction models to soybean hyperspectral point clouds to track the temporal dynamics of SPAD and EWT. Fig. 6 shows a sequence for the same potted plant from August 6 to August 21 at four wavelengths (552 nm, 662 nm, 702 nm, and 852 nm). We normalized the hyperspectral signals and mapped them onto the point clouds. For each date, different bands show distinct reflectance intensity distributions correlated with plant physiology. At 662 nm (red), the point cloud appears darker because leaves strongly absorb red light, reducing reflectance. In contrast, 552 nm (green) is brighter due to weaker chlorophyll absorption, and 702 nm (red-edge) and 852 nm (near-infrared) are bright because multiple scattering by leaf microstructures increases reflectance.

**Fig. 7:**
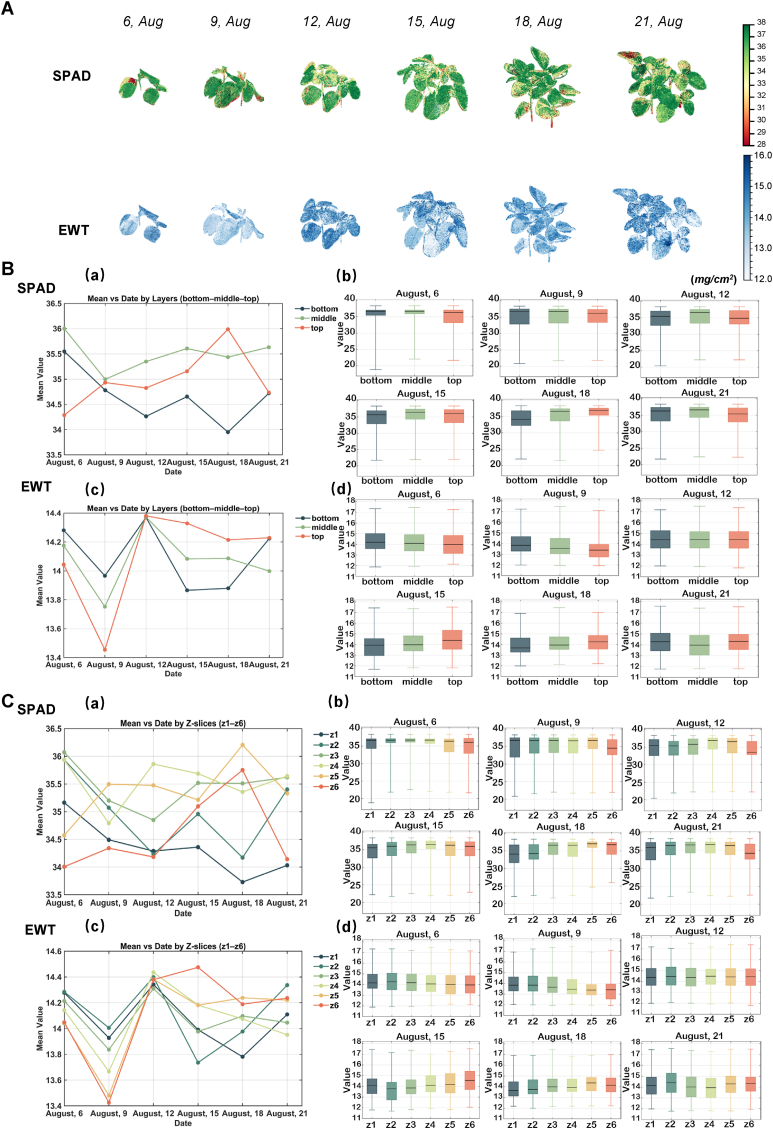
Temporal prediction results. A. Visualization of SPAD and EWT B. Temporal variation and distribution of SPAD and EWT across three vertical regions C. Temporal variation and distribution of SPAD and EWT across six vertical regions.

We applied the established prediction models for SPAD and EWT to the time-series data we collected. As illustrated in Fig. 7A, the spatial variations of SPAD and EWT from August 6 to August 21, 2025 can be clearly observed. As illustrated in the Fig. 7B, the soybean plants from each acquisition were spatially divided into top, middle, and bottom regions, and the SPAD and EWT values of these three regions were statistically analyzed to examine their spatial distribution. (a) and (c) respectively show the temporal variation curves of SPAD and EWT in the three regions. (b) and (d) display the daily distributions of SPAD and EWT across the three regions.

The temporal fluctuations of SPAD and EWT in the top, middle, and bottom regions were not pronounced, which may be due to the relatively short observation period. However, it is noteworthy that between August 9 and August 12, EWT exhibited a clear increase. This trend is also reflected in the point clouds in Fig. 7A: compared with August 9, the EWT point cloud of August 12 appears markedly darker, indicating an increase in EWT. This can be explained by the fact that the soybean pots were placed outdoors, and a period of heavy rainfall occurred on August 11, leading to a sharp rise in EWT. After August 12, EWT gradually declined as a result of transpiration. A certain degree of vertical variation was observed across the top, middle, and bottom regions. Within the same region, however, SPAD and EWT showed relatively scattered distributions. To further examine spatial variation, we subdivided the vertical space into six finer regions. As illustrated in Fig. 7C, this vertical stratification makes the differences even more evident, indicating that spatial heterogeneity of these two traits exists within the same plant. Our hyperspectral point clouds approach is capable of capturing such differences.

## 4 Discussion

### 4.1 Hyperspectral point clouds offer a new dimension for 3D plant phenotyping

Our results indicate that traditional 3D phenotyping mainly focuses on macroscopic parameters such as plant height or canopy volume, while overlooking the important physiological dimensions of plant traits. By integrating hyperspectral information into point clouds, we extended the capability of 3D phenotyping beyond structural features, providing a more comprehensive perspective that combines geometry and physiology. Through experiments on potted soybean plants, this method successfully linked hyperspectral features with SPAD and EWT traits, enabling their prediction and direct visualization in 3D space.

The spatial variation of SPAD and EWT across vertical canopy layers is an important observation. Although the overall temporal fluctuations were modest, canopy stratification revealed clear vertical heterogeneity, indicating that even within a single plant, trait distributions are not uniform. The ability of hyperspectral point clouds to capture such spatial heterogeneity demonstrates that combining spectral and structural dimensions can reveal subtle physiological patterns that might be overlooked by conventional 3D point cloud or 2D hyperspectral approaches.

From a broader perspective, this method highlights the potential of hyperspectral point clouds as a powerful tool for plant phenotyping. By enabling the joint exploration of structural and physiological traits in 3D, it opens up new opportunities for understanding plant function, monitoring stress responses. Future work may further explore its scalability toward field-scale phenotyping and the development of high-throughput hyperspectral point clouds generation and analysis.

### 4.2 Future perspectives of hyperspectral point clouds

In this study, trait prediction models were initially constructed using both hyperspectral information and the leaf normal vectors of soybean plants as input variables. The results showed that the contribution of leaf normal vectors to model performance was much lower than that of hyperspectral data. Even when only hyperspectral data were used, the prediction models for SPAD and EWT still achieved good results. This may be attributed to the large number and wide range of hyperspectral bands, which provide sufficient feature inputs for the model [49].

During hyperspectral data acquisition, a white reference panel was employed for spatial radiometric correction. However, due to the anisotropic reflectance arising from the non-Lambertian nature of plant surfaces, angular effects in hyperspectral measurements were unavoidable [50,51]. At close ranges, the white reference panel could not adequately characterize the light-field distribution, resulting in errors in spatial radiometric correction. In the subsequent modeling stage, standard normal variate (SNV) preprocessing was applied to the hyperspectral data to reduce scattering-related variability. An interesting observation is that, after SNV preprocessing, leaf angle information contributed only marginally to model performance. This suggests that SNV may alleviate angular-related variability in the hyperspectral data within the modeling framework.

Furthermore, we applied the prediction models to time-series hyperspectral point clouds and conducted a preliminary analysis of vertical canopy variation. The current analysis only considered vertical stratification of the canopy, with relatively coarse divisions, to observe basic spatial differences. Future 3D hyperspectral analyses are likely to focus more on fine-grained plant phenotypic traits, which will place higher demands on the accuracy of spatial radiometric correction. Previous studies have reported that, under controlled conditions, hemispherical references or physical compensation methods can be used to correct reflectance in close-range hyperspectral calibration [30,32], which holds great potential for building more precise hyperspectral point clouds. In future work, we will focus on constructing and analyzing higher-precision and finer-grained hyperspectral point clouds to gain deeper insights into the spatial heterogeneity of plant traits.

## 5 Conclusions

In this study, we developed and validated a framework for integrating hyperspectral information with 3D point clouds to advance plant phenotyping. By linking hyperspectral reflectance to SPAD and EWT traits, we established predictive models that enabled trait estimation and direct visualization within the 3D canopy. The results demonstrated that hyperspectral data alone provided strong predictive capacity, while leaf angular information contributed little, likely due to the effectiveness of SNV preprocessing in reducing angular effects.

Application of the models to time-series hyperspectral point clouds further revealed vertical spatial heterogeneity of SPAD and EWT within soybean canopies, highlighting the potential of this approach for exploring fine-scale physiological variation. Although our current analysis focused on relatively coarse vertical stratification and a limited observation period, the findings underscore the promise of hyperspectral point clouds as a powerful tool for capturing both structural and physiological dimensions of plant traits.

Future efforts will aim to refine the spatial and spectral resolution of hyperspectral point clouds, improve calibration under non-Lambertian conditions, and extend this framework to field-scale and high-throughput applications. Collectively, this study provides new insights into plant trait estimation and spatiotemporal analysis, and lays the foundation for more precise and comprehensive 3D phenotyping.

## Author contributions

Wenzhe Deng conceptualized the study and developed the methodology. Wenzhe Deng, Yajie Liu, and Zhichao Ni collected the dataset and performed data preprocessing. Wenzhe Deng wrote the original draft of the manuscript, while Wenzhe Deng, Tingting Wu, and Qingping Ling reviewed and edited it. Tingting Wu acquired the funding for the study. Tingting Wu supervised the project. All authors have approved the final version of the manuscript for submission.

## Declaration of competing interest

The authors declare that they have no known competing financial interests or personal relationships that could have appeared to influence the work reported in this paper.

## Data Availability

The datasets supporting the findings of this study are publicly available at https://github.com/wendell-deng/3DProjection.
